# Amiodarone for the Treatment of Arrhythmias in COVID-19 Patients Does Not Increase the Risk of Pulmonary Fibrosis: A Retrospective Cohort Study

**DOI:** 10.7759/cureus.34109

**Published:** 2023-01-23

**Authors:** David B Money, Dae Hyun Lee, Ari Hadar, Justin Doherty, Christopher Malanga, Alexa Serino, Adam J Cohen

**Affiliations:** 1 Department of Internal Medicine, University of South Florida, Tampa, USA; 2 Department of Cardiology, University of South Florida, Tampa, USA; 3 Department of Medicine, University of South Florida Morsani College of Medicine, Tampa, USA; 4 Department of Cardiology, University of South Florida Morsani College of Medicine, Tampa, USA

**Keywords:** renin-angiotensin-aldosterone system, cytokines, antiarrhythmics, dose-dependent toxicity, safety profile, pulmonary fibrosis, outcomes research, atrial fibrillation, covid-19, amiodarone

## Abstract

Amiodarone is a class III antiarrhythmic medication used to treat atrial and ventricular tachyarrhythmias. Pulmonary fibrosis from amiodarone use is a well-documented side effect. Pre-COVID-19 pandemic studies have shown that amiodarone-induced pulmonary fibrosis occurs in 1%-5% of patients and usually occurs between 12 to 60 months after initiation. The risk factors associated with amiodarone-induced pulmonary fibrosis include a high total cumulative dose (treatment longer than two months) and high maintenance dose (>400 mg/day). COVID-19 infection is also a known risk factor for developing pulmonary fibrosis and occurs in approximately 2%-6% of patients after a moderate illness. This study aims to assess the incidence of amiodarone in COVID-19 pulmonary fibrosis (ACPF). This is a retrospective cohort study with 420 patients with COVID-19 diagnoses between March 2020 and March 2022, comparing two populations, COVID-19 patients with exposure to amiodarone (N=210) and COVID-19 patients without amiodarone exposure (N=210). In our study, pulmonary fibrosis occurred in 12.9% of patients in the amiodarone exposure group compared to 10.5% of patients in the COVID-19 control group (p=0.543). In multivariate logistic analysis, which controlled for clinical covariates, amiodarone use in COVID-19 patients did not increase the odds of developing pulmonary fibrosis (odds ratio (OR): 1.02, 95% confidence interval (CI): 0.52-2.00). The clinical factors associated with the development of pulmonary fibrosis in both groups included a history of preexisting interstitial lung disease (ILD) (p=0.001), exposure to prior radiation therapy (p=0.021), and higher severity of COVID-19 illness (p<0.001). In conclusion, our study found no evidence that amiodarone use in COVID-19 patients increased the odds of developing pulmonary fibrosis at six-month follow-up. However, long-term amiodarone usage in the COVID-19 population should be based on the physician’s discretion.

## Introduction

Amiodarone is a class III antiarrhythmic medication utilized in the rhythm control of both atrial and ventricular tachyarrhythmias. Its mechanism works to prolong the action potential duration by blocking potassium channels. It also has beta-blocking properties and works on inactivated sodium channels to decrease ectopic automaticity within cardiac myocytes. Pulmonary fibrosis from amiodarone use is a well-documented side effect and typically occurs between 12 to 60 months after initiation [1]. Amiodarone-induced pulmonary fibrosis is associated with a high total cumulative dose (treatment greater than two months) and high maintenance dose (>400 mg/day). Given the extensive side effect profile of amiodarone, it is often reserved for patients with recurrent ventricular tachycardia, ventricular fibrillation, or atrial fibrillation with comorbid structural heart disease and heart failure [2,3].

Amiodarone and COVID-19 share similar pathophysiology toward the development of pulmonary fibrosis through the upregulation of the renin-angiotensin-aldosterone system (RAAS) and cytokines (interleukin (IL)-1, IL-8, tumor necrosis factor (TNF)-α, and transforming growth factor (TGF)-β) [4,5]. The risk factors for the development of pulmonary fibrosis in patients exposed to amiodarone (i.e., male gender, old age, and preexisting lung disease) have been well documented in the literature, including the Atrial Fibrillation Follow-up Investigation of Rhythm Management (AFFIRM) trial [6-12]. Amiodarone-induced lung fibrosis typically occurs with long-term exposure and high-maintenance daily dosing. Currently, the risk factors associated with COVID-19-induced pulmonary fibrosis include severe COVID-19 infection, preexisting idiopathic pulmonary fibrosis (IPF), older age, male sex, and comorbidities such as hypertension and diabetes [13,14]. Importantly, there is a lack of literature surrounding the safety of amiodarone use in COVID-19 patients [15]. Therefore, this study aims to assess if COVID-19 patients who were exposed to amiodarone were at a higher risk of developing pulmonary fibrosis.

## Materials and methods

Study design

**Figure 1:**
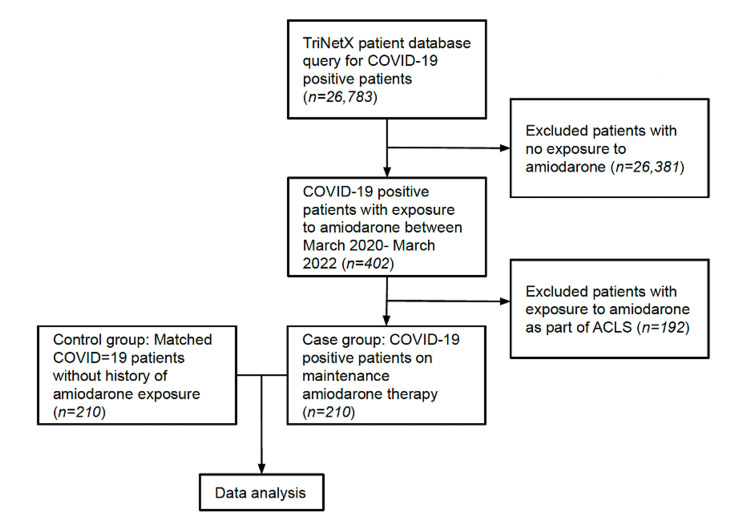
Study design COVID-19: coronavirus disease 2019, ACLS: advanced cardiovascular life support

This is a retrospective cohort study with a total of 420 patients divided into two groups: COVID-19 patients exposed to amiodarone (n=210) and COVID-19 patients without amiodarone use (n=210). Both groups were initially identified via database query through TriNetX of our institution at Tampa General Hospital (Tampa, FL, USA). Patients with COVID-19 (ICD code U07.1) were identified (N=26,783) between March 2020 and March 2022. The inclusion criteria for the COVID-19 and amiodarone exposure group included patients with current or previous amiodarone use (n=402). The exclusion criteria were exposure to amiodarone as part of short-term (<6 months) therapy or during the management of cardiac arrest (n=192) (Figure 1). The control group (COVID-19 without amiodarone use) was defined as positive for COVID-19 but without current or previous amiodarone use. The control group was matched to the case group on patient age, sex, and severity of COVID-19 illness. The severity of COVID-19 was defined by the National Institute of Health (NIH) as follows: mild illness, including upper respiratory symptoms; moderate illness with evidence of lower respiratory illness with no documented hypoxia (SpO2 > 94%); and severe illness with documented hypoxemia (SpO2 < 94%) on room air at sea level, PaO2/FiO2 < 300 mm Hg, respiratory rate > 30 breaths/minute, or lung infiltrates > 50%. To ensure COVID-19 severity was documented properly, we followed the guidelines set by the National Inpatient Sample (NIS) and national readmission database (NRD) COVID-19-related research [16]. For mild COVID-19 illness, we included patients with mild upper respiratory symptoms such as cough, low-grade fever, and anosmia. For moderate COVID-19, we incorporated ICD codes for pneumonia (ICD code J12.89), acute bronchitis (ICD code J20.8), and lower respiratory infection (ICD code J98.8), with exclusion criteria including hypoxemia and intensive care unit (ICU) level of care. Severe COVID-19 was found using ICD codes for acute respiratory distress syndrome (ICD code J80) and ICU level of care related to COVID-19 illness.

Outcome

The primary outcome of pulmonary fibrosis was assessed via chart review of each patient chart. All patients who met the criteria for the primary endpoint of pulmonary fibrosis were assessed on computed tomography (CT) (n=49). Radiographic features consistent with the diagnosis of pulmonary fibrosis on CT included reticulation, traction bronchiectasis, and fibrotic-appearing lung architectural distortions.

Statistical analysis

Data were presented as mean ± standard deviation or number (percentage) depending on the data type. We have performed univariate and multivariate logistic regression analyses. The univariate analysis (Model A) served as an unadjusted model that assessed the incidence of pulmonary fibrosis among the two groups without adjusting for clinical covariates. Model B assessed the incidence of pulmonary fibrosis among the two groups using logistic regression analysis that adjusted for patient age and sex. Model C is adjusted for patient age, sex, history of interstitial lung disease (ILD), history of chronic obstructive pulmonary disease (COPD), current or prior smoking, and severity of COVID-19 illness. Continuous variables were compared between groups using Student’s t-test. Categorical data were compared using chi-squared analysis. A two-tailed p-value of ≤0.05 was considered statistically significant. Statistical analysis was performed using R software version 4.0.4.

## Results

The baseline clinical characteristics of each group are shown in Table 1. The COVID-19 without amiodarone exposure control group was matched to the amiodarone exposure group on age in years (mean: 70.5 versus 69.3, p=0.368), sex (65.2% versus 66.2% males, p=0.918), and severity of COVID-19 (mild: 57.6% versus 55.7%, moderate: 14.8% versus 16.2%, severe: 27.6% versus 28.1%, p=0.898). The amiodarone exposure group had more individuals with a history of coronary artery disease (CAD) (40% versus 18.6%, p<0.001), a history of congestive heart failure (CHF) (47.1% versus 3.3%, p<0.001), and a history of asthma/COPD (23.3% versus 12.9%, p=0.008). The amiodarone exposure group also had significantly higher outpatient follow-up within one year of contracting COVID-19 (67.1% versus 39%, p<0.001). There was no significant difference between the groups regarding the number of ILD, rheumatoid arthritis (RA), systemic sclerosis (SS), sarcoidosis, systemic lupus erythematosus (SLE), and history of radiation exposure. In the amiodarone exposure group, there were significantly more patients who had maintenance amiodarone prescriptions for ≤200 mg compared to ≥400 mg (82.8% versus 17.2%, p<0.001) (Table 2). Regarding the temporal relationship of the two variables in the amiodarone exposure group, significantly more people had amiodarone exposure prior to contracting COVID-19 compared to new amiodarone prescriptions after contracting COVID-19 (64.3% versus 35.7%, p<0.001). Atrial fibrillation was the predominant indication for amiodarone prescription at 84.3%, followed by ventricular tachycardia at 11.4% and supraventricular tachycardia at 4.3% (Table 3).

**Table 1:** Baseline clinical characteristics COVID-19, coronavirus disease 2019; TFT, thyroid function test; TSH, thyroid-stimulating hormone; BMI, body mass index; ILD, interstitial lung disease; COPD, chronic obstructive pulmonary disease; SLE, systemic lupus erythematosus; RA, rheumatoid arthritis; SS, systemic sclerosis

	COVID-19 (n=210)	COVID-19 + amiodarone (n=210)	p-value
Age (years)	70.5±12.9	69.3±13.7	0.368
Gender			0.918
Male	137 (65.2%)	139 (66.2%)	
Female	73 (34.8%)	71 (33.8%)	
TFT			0.001
TSH < 0.5 mIU/L	8 (3.8%)	20 (9.5%)	
TSH = 0.5-5 mIU/L	200 (95.2%)	178 (84.8%)	
TSH > 5 mIU/L	2 (1%)	12 (5.7%)	
BMI (kg/m^2^)	28.2±6.3	29±7.9	0.26
Coronary artery disease	39 (18.6%)	84 (40%)	<0.001
Congestive heart failure	7 (3.3%)	99 (47.1%)	<0.001
Interstitial lung disease	4 (1.9%)	6 (2.9%)	0.749
Asthma/COPD	27 (12.9%)	49 (23.3%)	0.008
SLE, RA, SS, sarcoidosis	5 (2.4%)	6 (2.9%)	1
Radiation	3 (1.4%)	3 (1.4%)	1

**Table 2:** Outcomes-related data *Mild COVID-19, upper respiratory symptoms; moderate COVID-19, evidence of lower respiratory illness with no documented hypoxia (SpO2 > 94%); severe COVID-19, documented hypoxemia (SpO2 < 94%) on room air at sea level, PaO2/FiO2 < 300 mm Hg, respiratory rate > 30 breaths/minute, or lung infiltrates > 50% COVID-19, coronavirus 2019

	COVID-19 (n=210)	COVID-19 + amiodarone (n=210)	p-value
Patients with outpatient follow-up within one year			<0.001
Yes	82 (39%)	141 (67.1%)	
Pulmonary fibrosis at six months	22 (10.5%)	27 (12.9%)	0.543
Severity of COVID-19*			0.898
Mild	121 (57.6%)	117 (55.7%)	
Moderate	31 (14.8%)	34 (16.2%)	
Severe	58 (27.6%)	59 (28.1%)	

**Table 3:** Amiodarone use characteristics *Amiodarone load defined as intravenous or oral administration of high daily dosages (typically a total of 6-10 g) prior to maintenance dosing COVID-19, coronavirus 2019

	Percentage of patients in amiodarone exposure group (n=210)
Amiodarone load*	107 (51%)
Temporal relationship of exposure	
COVID-19 before amiodarone	75 (35.7%)
Amiodarone before COVID-19	135 (64.3%)
Maintenance amiodarone dose (mg) per day	
≤200 mg	174 (82.9%)
>200 mg	36 (17.1%)
Indication for amiodarone	
Atrial fibrillation	177 (84.3%)
Supraventricular tachycardia	9 (4.3%)
Ventricular tachycardia	24 (11.4%)

**Figure 2:**
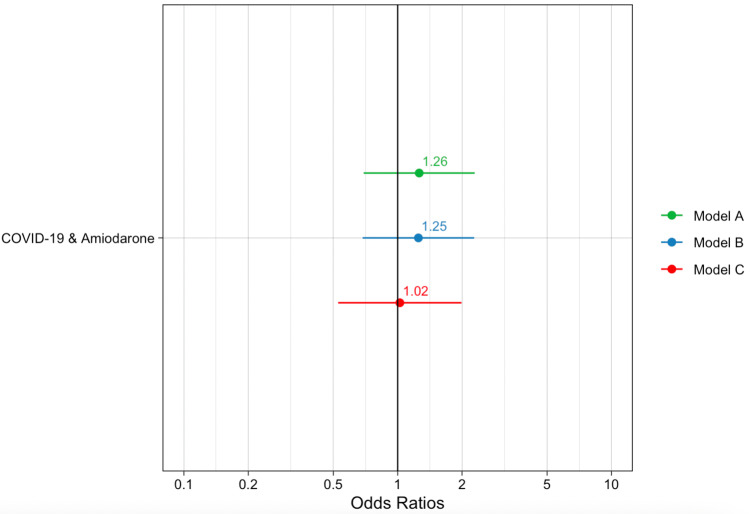
Summary of risk of pulmonary fibrosis in COVID-19 patients exposed to amiodarone at six-month follow-up OR plot comparing the incidence of pulmonary fibrosis between the amiodarone and COVID-19 exposure group and the COVID-19 without amiodarone exposure group Model A, unadjusted model; Model B, adjusted for age and sex; Model C, adjusted for age, sex, history of interstitial lung disease and chronic obstructive lung disease, prior smoking history, and severity of COVID-19 illness COVID-19, coronavirus 2019; OR: odds ratios

Pulmonary fibrosis occurred in 12.9% of patients in the amiodarone and COVID-19 exposure group compared to 10.5% of patients in the COVID-19 control group (p=0.543) (Table 4). The clinical factors associated with pulmonary fibrosis included a history of ILD (p=0.001), a history of exposure to radiation therapy (p=0.021), and higher severity of COVID-19 (p<0.001). In the univariate analysis, there was no statistically significant difference in pulmonary fibrosis outcome in COVID-19 patients who took amiodarone compared to control (odds ratio (OR): 1.26, 95% confidence interval (CI): 0.69-2.29). In Model B, we adjusted for patient age and sex and found no significant difference in the incidence of pulmonary fibrosis in COVID-19 patients exposed to amiodarone (OR: 1.25, 95% CI: 0.69-2.28). In Model C, by controlling for patient age, sex, history of ILD, COPD, smoking, and severity of COVID-19, we again found that amiodarone use in COVID-19 patients did not increase the odds of developing pulmonary fibrosis compared to COVID-19 patients without exposure to amiodarone (OR: 1.02, 95% CI: 0.53-1.99) (Figure 2). Lastly, there was no evidence of a difference in pulmonary fibrosis when controlling eight different medications known to cause pulmonary toxicity (Table 5).

**Table 4:** Descriptive statistics based on pulmonary fibrosis outcome and several clinical covariates *Mild COVID-19, upper respiratory symptoms; moderate COVID-19, evidence of lower respiratory illness with no documented hypoxia (SpO2 > 94%); severe COVID-19, documented hypoxemia (SpO2 < 94%) on room air at sea level, PaO2/FiO2 < 300 mm Hg, respiratory rate > 30 breaths/minute, or lung infiltrates > 50% COVID-19, coronavirus 2019; TFT, thyroid function test; TSH, thyroid-stimulating hormone; BMI, body mass index; ILD, interstitial lung disease; COPD, chronic obstructive pulmonary disease; SLE, systemic lupus erythematosus; RA, rheumatoid arthritis; SS, systemic sclerosis

	No pulmonary fibrosis (n=371)	Pulmonary fibrosis (n=49)	p-value
Amiodarone use			
COVID-19 only	188 (50.7%)	22 (44.9%)	0.543
COVID-19 + amiodarone	183 (49.3%)	27 (55.1%)	
Age (years)	70.1±13.3	68.8±13.3	0.525
Gender			0.169
Male	239 (64.4%)	37 (75.5%)	
Female	132 (35.6%)	12 (24.5%)	
Patients with outpatient follow-up within six months	194 (52.3%)	29 (59.2%)	0.449
TFT			0.003
TSH < 0.5 mIU/L	22 (5.9%)	6 (12.2%)	
TSH = 0.5-5 mIU/L	340 (91.6%)	38 (77.6%)	
TSH > 5 mIU/L	9 (2.4%)	5 (10.2%)	
BMI (kg/m^2^)	28.7±7.2	27.6±7.0	0.299
Severity of COVID-19*			<0.001
Mild	230 (62%)	8 (16.3%)	
Moderate	54 (14.6%)	11 (22.4%)	
Severe	87 (23.5%)	30 (61.2%)	
Coronary artery disease	106 (28.6%)	17 (34.7%)	0.473
Congestive heart failure	95 (25.6%)	11 (22.4%)	0.762
History of ILD	5 (1.3%)	5 (10.2%)	0.001
Asthma/COPD	63 (17%)	13 (26.5%)	0.151
SLE, RA, SS, sarcoidosis history	8 (2.2%)	3 (6.1%)	0.247
Radiation history	3 (0.8%)	3 (6.1%)	0.021
Duration of amiodarone exposure			1.00
≤2 years	127 (69.4%)	19 (70.4%)	
>2 years	56 (30.6%)	8 (29.6%)	

**Table 5:** Medications with risk of pulmonary fibrosis

Medication	No pulmonary fibrosis (n=371)	Pulmonary fibrosis (n=49)	p-value
Procainamide	4 (1.1%)	0 (0%)	1
Methotrexate	8 (2%)	1 (2.2%)	1
Cyclophosphamide	1 (0.3%)	0 (0%)	1
Nitrofurantoin	20 (5.4%)	5 (10.2%)	0.309
Bleomycin	1 (0.3%)	0 (0%)	1
Busulfan	0 (0%)	0 (0%)	1
Carmustine	0 (0%)	0 (0%)	1
Penicillamine	0 (0%)	0 (0%)	1

## Discussion

Our study suggests that amiodarone exposure in COVID-19 patients does not increase the risk of pulmonary fibrosis compared to COVID-19 patients who are not exposed to amiodarone. This was contrary to our initial hypothesis where we postulated that dual exposure to amiodarone and COVID-19 would significantly increase the risk of developing pulmonary fibrosis. As expected, the incidence of pulmonary fibrosis in COVID-19 patients exposed to amiodarone was higher than pre-COVID-19 pandemic estimates of amiodarone-induced toxicity (12.9% versus 1%-5%) [17-20]. In subgroup analysis, several variables were associated with higher odds of developing pulmonary fibrosis in both groups. This included a higher severity of COVID-19 illness, a history of preexisting ILD, and a history of radiation therapy. A history of underlying COPD was not a predisposing factor in both the amiodarone exposure and COVID-19-only groups, contrary to a meta-analysis in May 2022 [21]. Furthermore, underlying CAD, CHF, or autoimmune disorders (i.e., SLE and RA) did not predispose COVID-19 patients toward developing pulmonary fibrosis when exposed to amiodarone. Within the amiodarone exposure group, there was no association with concurrent use of medications associated with the development of pulmonary fibrosis and amiodarone (Table 5).

This study has significant clinical implications for clinicians prescribing amiodarone in the post-COVID-19 era. It is essential to note that COVID-19 is an independent risk factor for the development of atrial fibrillation, with a documented incidence of 19%-21% compared to 1%-4% in the general population [22]. Thus, COVID-19 patients represent a patient cohort with a higher incidence of atrial fibrillation and potentially higher demand for amiodarone prescriptions in special patient populations (i.e., recurrent ventricular tachycardia, ventricular fibrillation, or atrial fibrillation with comorbid structural heart disease and heart failure). Based on our study results, clinicians can prescribe amiodarone to COVID-19 patients without increasing the odds of developing pulmonary fibrosis. Ultimately, the decision to start amiodarone in COVID-19 patients should be at the discretion of the prescribing clinician, with consideration for other rhythm control modalities.

Several limitations exist within our study. Firstly, the follow-up period from COVID-19 illness to the diagnosis of pulmonary fibrosis is relatively short, primarily driven by the retrospective nature of our study. In future studies, a prospective study design that assesses pulmonary fibrosis multiple years after COVID-19 diagnosis would likely capture the true incidence of pulmonary fibrosis in COVID-19 patients exposed to amiodarone. The recency of the COVID-19 pandemic prohibited such a prospective design for the study period of March 2020-March 2022. Secondly, the small number of pulmonary fibrosis cases (n=49) limits the statistical power to analyze, especially in subgroup analysis, risk factors that could predispose to pulmonary fibrosis in COVID-19 patients prescribed amiodarone. Although there was no evidence that asthma/COPD, obesity, CAD, or CHF increased the risk of pulmonary fibrosis in COVID-19 patients prescribed amiodarone, a larger prospective study would likely improve the statistical power for further analysis of these clinical variables.

Additionally, the percentage of patients with pulmonary fibrosis in our COVID-19 control group (10.5%) is higher than the reported incidence in the literature of moderate COVID-19 illness (2%-6%) [23]. This either suggests that current estimates of pulmonary fibrosis in COVID-19 patients are underestimated in the literature or that our COVID-19 control was sicker than the actual COVID-19 population. Lastly, we understand the inherent bias of reliance on electronic medical record review. This includes, but is not limited to, potential inconsistency in consistent medical documentation from clinicians and proper ICD documentation by medical coders.

## Conclusions

To the best of our knowledge, this study is the first to investigate the risk of pulmonary fibrosis with amiodarone use in COVID-19 patients. We were able to conclude that amiodarone exposure does not increase the short-term incidence of pulmonary fibrosis in COVID-19 patients. Future prospective studies are warranted to assess the overall incidence of pulmonary fibrosis in COVID-19 patients exposed to amiodarone. In conclusion, our study did not find any association that amiodarone use in COVID-19 patients increased the risk of pulmonary fibrosis.
